# Regenerative therapies for myocardial infarction: exploring the critical role of energy metabolism in achieving cardiac repair

**DOI:** 10.3389/fcvm.2025.1533105

**Published:** 2025-02-07

**Authors:** Jiahao Ren, Xinzhe Chen, Tao Wang, Cuiyun Liu, Kun Wang

**Affiliations:** ^1^ Key Laboratory of Maternal & Fetal Medicine of National Health Commission of China, Shandong Provincial Maternal and Child Health Care Hospital Affiliated to Qingdao University, Jinan, China; ^2^ Jinan Microecological Biomedicine Shandong Laboratory, Jinan, China

**Keywords:** myocardial infarction, cardiomyocyte proliferation, cardiac regeneration, energy metabolism, cell cycle

## Abstract

Cardiovascular diseases are the most lethal diseases worldwide, of which myocardial infarction is the leading cause of death. After myocardial infarction, in order to ensure normal blood supply to the heart, the remaining cardiomyocytes compensate for the loss of cardiomyocytes mainly by working at high capacity rather than by proliferating to produce new cardiomyocytes. This is partly due to the extremely limited ability of the adult heart to repair itself. A growing body of research suggests that the loss of cardiac regenerative capacity is closely related to metabolic shifts in energy sources. Currently, a large number of studies have focused on changes in metabolic levels before and after the proliferation window of cardiomyocytes, so it is crucial to search for relevant factors in metabolic pathways to regulate the cell cycle in cardiomyocyte progression. This paper presents a review of the role of myocardial energy metabolism in regenerative repair after cardiac injury. It aims to elucidate the effects of myocardial metabolic shifts on cardiomyocyte proliferation in adult mammals and to point out directions for cardiac regeneration research and clinical treatment of myocardial infarction.

## 1 Introduction

Myocardial infarction (MI) remains a significant contributor to morbidity, but mortality has been significantly reduced in the last decades due to the introduction of thrombolytic therapy and acute vascular interventions (1). Despite the enormous global efforts in the prevention and treatment of MI, the situation faced remains critical, partly because adult mammalian cardiomyocytes are at the stage of terminal differentiation and have an extremely limited proliferative capacity. Cardiomyocytes die under the action of persistent cardiac ischemia and other injurious factors, and irreversible damage occurs to the heart, which is one of the important mechanisms causing MI (2). At this stage, the most important strategy for the treatment of MI is the rapid restoration of blood flow. Reperfusion therapies, such as coronary intervention, are an important means of treating MI in a timely and effective manner to minimize cardiac damage. However, when blood supply is restored, it triggers ischemia-reperfusion injury (I/R), which leads to a series of serious pathological sequelae and further aggravates cardiac damage (3). MI progresses to the final stage leading to the development of heart failure (HF), and heart transplantation is the most effective therapeutic strategy for patients with end-stage HF, but organ shortages are the main problem faced today. Currently, commonly used clinical agents include statins, receptor blockers, or angiotensin-converting enzyme (ACE) inhibitors, but the search for new therapeutic targets remains an important endeavor. Therefore, the search for novel preventive and curative measures for MI is of great significance in reducing cardiovascular disease mortality.

In contrast to the limited self-repair capacity of the adult heart, the heart of newly born mice has a strong regenerative capacity, and the proliferation of the newly formed cardiac tissue comes mainly from the proliferation of endogenous cardiomyocytes (4). When MI occurs in the adult heart, mature cardiomyocytes do not have the ability to divide and proliferate; instead, the surrounding healthy cells make up for the missing parts by proliferation. However, this is not enough to replace the dead cardiomyocytes, which will cause myocardial ischemia and hypoxia, and ultimately lead to myocardial degeneration, necrosis, fibrosis, etc (5). The decreased proliferative capacity of cardiomyocytes may be related to the loss of myocardial progenitor cells or the reduced proliferative potential of mature cardiomyocytes during cardiac development (6). Recently, several studies have aimed to repair the heart by inducing cardiomyocyte re-entry into the cell cycle and promoting cardiomyocyte proliferation after MI (7). Thus, stimulating the proliferation of terminally differentiated cardiomyocytes and promoting cardiac regeneration is a viable approach for post-MI therapy.

In recent years, researchers have found that the mammalian heart converts its major metabolic substrate from glucose to fatty acids shortly after birth. This corresponds to a change in metabolic mode from anaerobic glycolysis to oxidative phosphorylation. Importantly, this metabolic conversion occurs concurrently with the loss of cardiac regenerative capacity (8). Thus, the loss of cardiac regenerative capacity in adults is associated with a shift in energy metabolism. The metabolic shift from glycolysis to oxidative phosphorylation leads to a stagnation of the cell cycle in cardiomyocytes, resulting in the loss of cardiac regenerative capacity (8–10). In summary, it is crucial to understand the metabolic changes in the heart before and after the proliferative window in mammals. It is also important to promote the re-entry of mature cardiomyocytes into the cell cycle by inducing metabolic pathway-related factors. This can provide new research directions in the field of promoting cardiomyocyte proliferation and cardiac regeneration.

In this paper, we briefly review the changes in mitochondrial energy metabolism during cardiac regeneration, focusing on the application of sugar metabolism, especially the glycolytic process, in regenerative repair after cardiac injury. We further summarize the strategies for cardiac regenerative repair after MI by targeting cardiomyocytes’ glucose metabolism, fatty acid metabolism, amino acid metabolism, ketone body metabolism, and tricarboxylic acid cycle metabolism, which provides insights into potential therapeutic approaches.

## 2 Energy metabolism in the healthy heart

**Figure 1:**
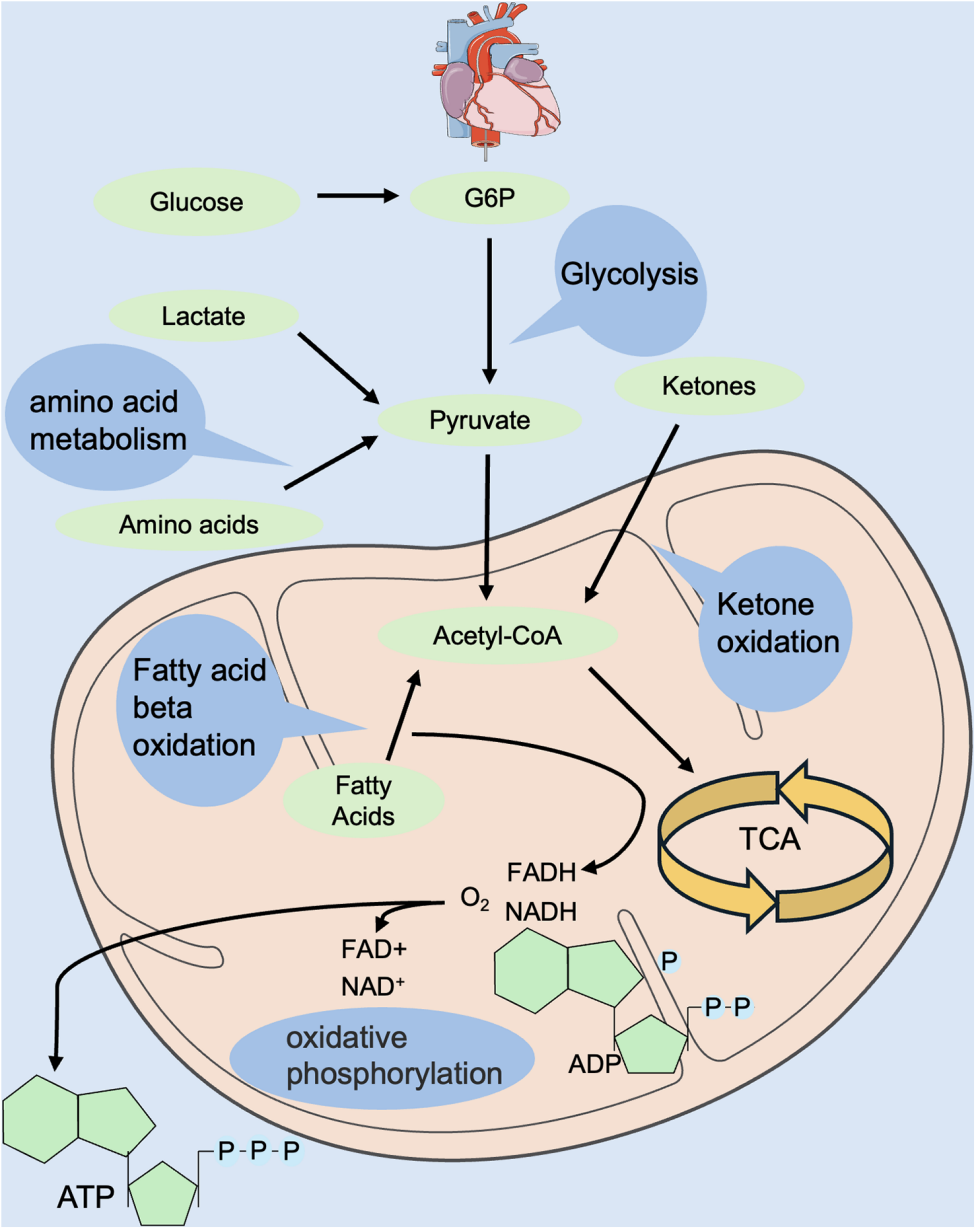
Regulation of energy metabolism in the normal heart. Energy metabolism includes glycolysis, fatty acid oxidation, amino acid oxidation, ketone body oxidation, tricarboxylic acid cycle, oxidative phosphorylation, etc. Energy donors include glucose, fatty acids, amino acids, ketone bodies, etc. Hydrogen donors include NADH and FADH. FADH, flavin adenine dinucleotide; NADH, reduced nicotinamide adenine dinucleotide.

The heart is the top energy-consuming organ in the body, but its energy reserves are low. The energy to maintain normal pump function and basal metabolism in the adult heart is mainly derived from mitochondrial oxidative phosphorylation and glycolysis (11, 12). Glycolysis produces ATP without the involvement of oxygen, and compared with glucose, fatty acid oxidation consumes more oxygen to be utilized by the electron transport chain for oxidative phosphorylation. Oxidative phosphorylation derives 40%–60% of its energy from fatty acid oxidation and glucose metabolism (13, 14), but these substrates have limited storage capacities. Therefore, the selection of suitable substrates for energy generation, as well as the maintenance of a dynamic network balance of energy metabolism, is crucial (15) (Figure 1).

During the mammalian embryonic period, the heart is in a low-oxygen environment and requires energy mainly from glycolysis and lactate metabolism and, to a lesser extent, from oxidative phosphorylation (16–18). Nearly half of the ATP required by the heart in neonatal suckling rats (P7) is supplied by glycolysis (16), whereas after P7, the utilization of glycolysis gradually decreases and is replaced by a significant increase in fatty acid oxidation to maintain the energy metabolism of the heart (16, 19). During the juvenile period, the energy source of the heart is mainly fatty acid oxidation, and the amount of ATP produced almost approaches that of the adult mammalian heart (20). In conclusion, oxygen levels at different developmental stages regulate changes in cardiac metabolic energy, which in turn affects cardiomyocyte metabolism and ultimately regulates cardiomyocyte proliferative capacity.

After cardiac injury, especially in the stage of HF, the metabolic processes of glucose and fatty acids in cardiomyocytes are disturbed. This disruption leads to alterations in the metabolic state of cardiomyocytes and remodeling of myocardial energy metabolism, which ultimately results in abnormalities in myocardial structure and function (21). On the one hand, during this process, substrate utilization is altered, and myocardial energy supply is converted from fatty acid oxidation to glycolysis, resulting in “embryonic re-evolution” of energy metabolism (22). On the other hand, mitochondrial structural abnormalities, reduced respiratory chain complex activity and ATP synthase activity, and impaired oxidative phosphorylation in HF lead to reduced energy production and cardiomyocyte damage (23–26).

In summary, energy metabolism in the healthy adult heart is highly flexible. Under normal conditions, the metabolic substrate is primarily fatty acid oxidation. When the heart is injured, it undergoes metabolic remodeling. This involves disturbances in glucose and fatty acid metabolism, as well as abnormalities in myocardial structure and function. In addition to a change in substrate, there is a shift from fatty acid oxidation to glycolysis, along with a series of changes in the metabolism of cardiomyocytes.

## 3 Metabolic patterns regulate cardiac regeneration

It was long believed that highly differentiated cardiomyocytes permanently exit the cell cycle until Porrello et al. (4) found that the neonatal suckling mouse heart has the potential to regenerate. In addition, a growing number of studies have shown that cardiomyocytes can be reintroduced into the cell cycle by stimulating them in adult mammals (27–29). Recently, metabolism-related factors have been recognized as key factors regulating cardiomyocyte cell cycle progression.

### 3.1 Metabolic shift from glycolysis to oxidative phosphorylation inhibits cardiomyocyte proliferation

**Figure 2:**
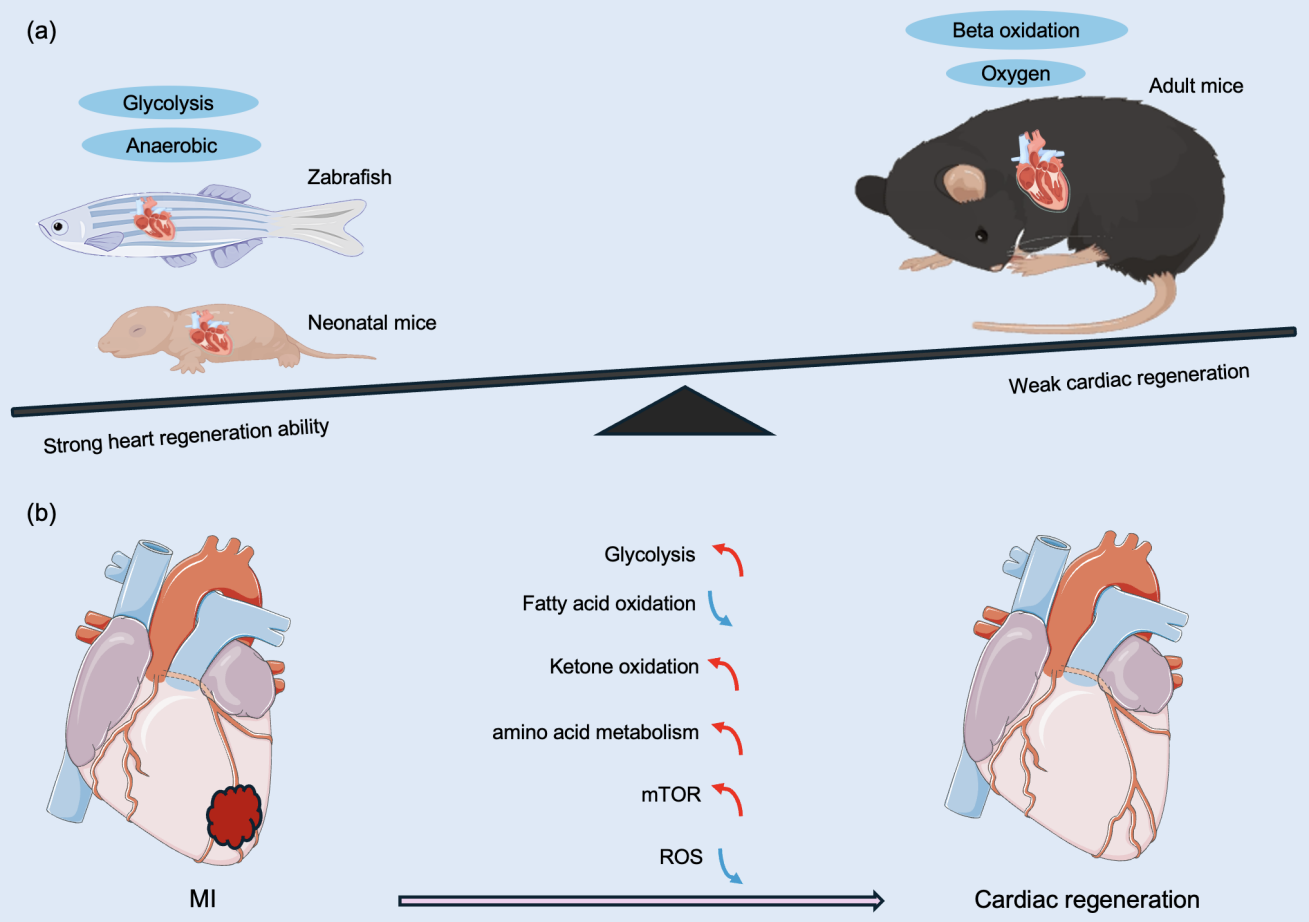
Different metabolic patterns before and after birth affect myocardial regeneration capacity. **(a)** Comparison of regenerative capacity and energy metabolism on regeneration of damaged hearts after MI in representative lower vertebrates, neonatal animals, and adult mammals. **(b)** Anaerobic glycolysis, ketone body oxidation, amino acid metabolism, and the mTOR pathway promote cardiac regeneration and improve regenerative capacity. Changes in cardiac metabolic state during neonatal mouse growth, fatty acid β-oxidation under aerobic conditions, and ROS lead to a decrease in myocardial regenerative capacity. MI, myocardial infarction; mTOR, mechanistic target of rapamycin; ROS, reactive oxygen species. The red line indicates activation, the blue line indicates inhibition, the green line indicates a decrease in level, and the purple line indicates an increase in level.

Low-vertebrate animals, such as zebrafish, maintain a strong regenerative capacity of the heart in adulthood (30–32). In 2002, Poss et al. (30) utilized surgical removal of 20% of the heart of an adult zebrafish, and the full regeneration of the zebrafish heart was achieved 60 days after the surgery. Subsequently, a zebrafish cardiac cryoinjury model was established to simulate mammalian MI, and in the following two months, the fibrotic scar tissue was gradually eliminated by apoptosis and eventually replaced by newborn myocardium (33). To investigate whether the mechanism of cardiomyocyte regeneration after the specific killing of cardiomyocytes is the same as that after mechanical injury, a genetic cell-killing model was established in zebrafish. In this model, cardiomyocytes rapidly proliferated and completed regeneration in the following days (34). Similar to zebrafish, 1-day-old neonatal mice also achieve full regeneration after cardiac injury (4). However, in 7-day-old mice subjected to apicoectomy, there was no regenerating myocardium in the area of injury, and irreversible fibrosis occurred, with the heart losing its regenerative capacity (4). So what prevents the proliferation and regeneration of cardiomyocytes in mammals such as mice seven days after birth (Figure 2)?

Adult zebrafish and mammals have been exposed to a hypoxic environment during embryonic life, and the heart has a strong regenerative capacity (35). In contrast, mammals are exposed to a hypoxic to a hyperoxic environment after birth, which corresponds to a marked difference in energy metabolism between the embryonic and adult heart. During embryonic development, cardiomyocytes use anaerobic glycolysis as the main source of energy, whereas adult cardiomyocytes primarily utilize oxygen-dependent mitochondrial oxidative phosphorylation for energy supply (8, 36). In addition, mature cardiomyocytes derive more than 80% of their energy from fatty acid β-oxidation (37), and oxidative phosphorylation produces 18 times more ATP than cytoplasmic glycolysis (19). Although oxidative phosphorylation has a greater energetic advantage over glycolysis, it is accompanied by an increased accumulation of reactive oxygen species (ROS), resulting in an increase in the DNA damage response pathway (DDR) pathway (38), which is one of the factors that induce cardiomyocytes to exit the cell cycle. Mitochondrial ROS are generated by the leakage of electrons from the mitochondrial respiratory chain and the reaction of these electrons with O2 (39, 40), which can cause oxidative damage to DNA, single- or double-strand breaks, and ultimately cell cycle arrest (41).

It has been shown that after birth in mammals, the expression of most of the enzymes associated with glycolysis decreases significantly after seven days of life, while most of the enzymes involved in the mitochondrial Krebs cycle are upregulated within the same timeframe (42). This is consistent with the pattern of the cell cycle in cardiomyocyte arrest. Thus, the postnatal shift in metabolic pattern from glycolysis to oxidative phosphorylation results in the inhibition of the cell cycle in cardiomyocyte progression.

### 3.2 Metabolic reprogramming regulates cardiomyocyte cell proliferation and cardiac regeneration

The adult mouse heart renews approximately 5% of its cardiomyocytes annually, with the majority located in the subendocardial muscle (43–45). Furthermore, in the human heart, cardiomyocyte proliferative capacity is around 1% at age 20 and decreases to 0.4% at age 75 (46–48). In contrast, the neonatal mammalian heart has a potential regenerative capacity; however, this regenerative capacity is lost after the first week of life (4, 49), which is closely related to the metabolic shift from glycolysis to oxidative phosphorylation in cardiomyocytes after birth (8–10, 50, 51). This is because increased mitochondrial oxidative phosphorylation activity promotes cardiomyocyte maturation while decreasing the proliferative capacity of cardiomyocytes (52).

Under pathological conditions such as MI or HF, cardiomyocytes revert from glucose as an energy source (53). This is mainly due to the fact that after cardiomyocyte ischemia, oxygen concentration decreases, and energy metabolism is forced to switch to glycolysis, resulting in the reentry of cardiomyocytes into the cell cycle and reactivation of cardiomyocyte proliferation in the injured heart (52, 54, 55).

Taken together, the metabolic pathway shift from anaerobic glycolysis in fetal life to oxidative phosphorylation in adulthood inhibits the proliferative capacity of mammalian cardiac cardiomyocytes.

## 4 Energy metabolism in cardiac regeneration

**Figure 3:**
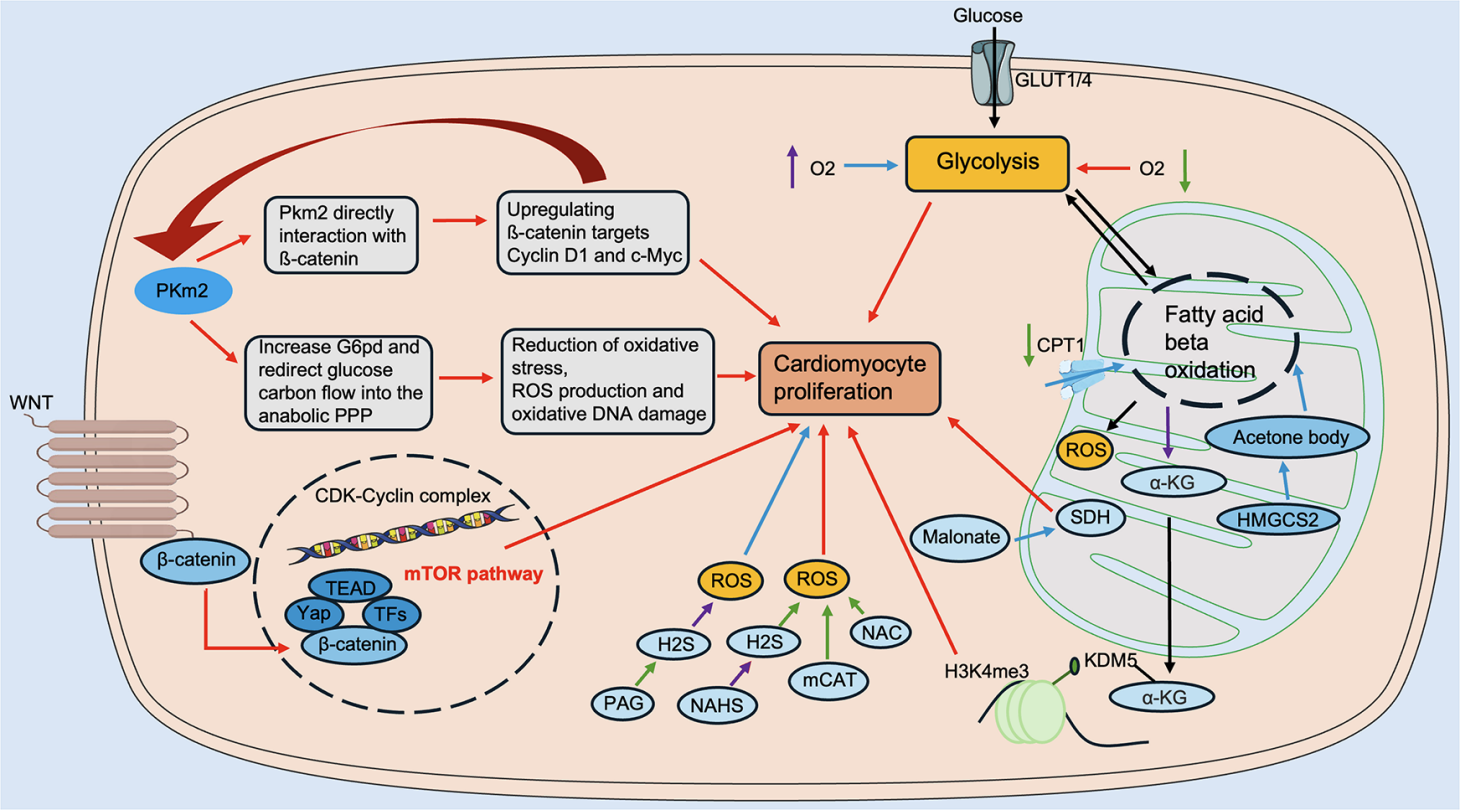
Regulation of energy metabolism in cardiac regeneration. Glucose metabolism includes Pkm2, Malonate, SDH, etc. Fatty acid metabolism includes CPT1, α-KG, KDM5, and others. Amino acid metabolism includes Wnt/β-catenin, mTOR, and others. Ketone body metabolism includes HMGCS2, etc. ROS modulators include NAHS, mCAT, NAC, PAG, etc. α-KG, α-ketoglutarate; CPT1, carnitine palmitoyltransferase 1; HMGCS2, 3-hydroxy-3-methylglutaryl-CoA synthase 2; KDM5, lysine demethylase 5; mCAT, mitochondrial peroxiredoxin; mTOR, mechanistic target of rapamycin; NAHS, sodium hydrosulfide hydrate; NAC, N-acetylcysteine; PAG, DL-Propargylglycine; Pkm2, pyruvate kinase muscle isoform 2; ROS, reactive oxygen species; SDH, succinate dehydrogenase. The red line indicates activation, the blue line indicates inhibition, the green line indicates a decrease in level, and the purple line indicates an increase in level.

In recent years, changes in the metabolic levels of the heart in pathological states have received increasing and widespread attention. The metabolic level of the heart is highly regulated, allowing for the ability to switch between available substrates. It is well known that fatty acids are the energy substrates with the lowest energy production efficiency (production of ATP/consumption of O2) (53). In contrast, glucose, an important fuel in the heart, is the most energy-efficient substrate for ATP production under anaerobic conditions via pyruvate conversion by glycolysis (53). In recent years, lactate metabolism, amino acid oxidation, and ketone body metabolism have also been recognized as potential sources of cardiac energy (56–60). In the following, we provide a brief overview of energy metabolism during cardiac regeneration (Figure 3).

### 4.1 Glucose metabolism in cardiac regeneration

Glucose is an important energy substrate in the heart. A growing body of research now suggests a potential link between glucose metabolism and cardiomyocyte proliferation. Glucose is transported into cardiomyocytes via glucose transporter proteins (GLUT), of which GLUT1 is the predominant isoform in mammalian embryonic and neonatal hearts (61), which corresponds to the fact that glycolysis is embryonic and neonatal. Studies have shown that when GLUT1 is overexpressed, there is an increase in glucose uptake in cardiomyocytes and an increase in glycogen accumulation, and the metabolic pattern shifts to glycolysis, promoting cardiomyocyte proliferation (62). In addition, flow cytometry identified two distinct cell populations: a recombinant troponin t type (Tnnt)1^high^ cell population and a relatively immature Tnnt2^low^ cell population, in which the Tnnt2^low^ cell population responds to high glucose uptake after cardiac injury and promotes mitosis in cardiomyocytes, thereby accelerating cardiac regeneration (62).

Key components of glycolysis play an integral role in cardiac regeneration. Pyruvate dehydrogenase kinase (PDK) is an important enzyme in the regulation of glycolysis, and the expression level of PDK is significantly up-regulated in both zebrafish and mammalian injured hearts and regulates cardiomyocyte proliferation in the injured heart (54). Pyruvate kinase muscle isoform 2 (PKM2), a key enzyme in the final step of glycolysis, promotes cardiomyocyte cell division by regulating the cell cycle through the anabolic pathway and β-catenin and reducing oxidative stress injury. After MI, PKM2 induces cardiomyocyte cell cycle, increases angiogenesis, prolongs cardiomyocyte survival time, and reduces cardiomyocyte apoptosis and oxidative stress to prevent or reverse cardiac remodeling after MI (63). Therefore, targeting the cell cycle to promote cardiomyocyte proliferation and cardiac regeneration is an effective approach.

In summary, glucose metabolism plays a critical role in cardiac regeneration. Although more research is needed to elucidate myocardial regeneration in greater detail, these findings provide hope that targeting one or more key components of glucose metabolism in the clinical setting may be one of the most effective strategies for cardiac regeneration and functional recovery.

### 4.2 Fatty acid metabolism in cardiac regeneration

During the embryonic period, cardiomyocytes are virtually not able to utilize fatty acids for energy production due to limited mitochondrial and fatty acid availability. After birth, the fetal heart rapidly switches to fatty acid β-oxidation (64, 65), a change that coincides with the point in time when cardiac regenerative capacity is lost. Thus, fatty acid metabolism is closely related to cardiomyocyte proliferation.

It has been shown that postnatal neonatal suckling rats consuming milk deficient in fatty acids prolonged the cardiomyocyte proliferation window (66). Thus, inhibition of fatty acid oxidation promoted myocardial proliferation and regeneration. Carnitine palmitoyltransferase (CPT), as a key enzyme in fatty acid oxidation, plays an essential role in regulating cardiomyocyte proliferation. The relative activity of CPT1 in cardiomyocyte mitochondria increases progressively after birth in mice (67), whereas the use of an inhibitor of CPT1 results in a decrease in fatty acid β-oxidation and enhances cardiomyocyte proliferation (68, 69). Inhibition of fatty acid oxidation through modulation of CPT1 isoforms results in the accumulation of α-ketoglutarate (α-KG). This, in turn, activates the α-ketoglutarate-dependent lysine demethylase KDM5. The activated KDM5 then drives the demethylation of the mature H3K4me3 structural domain in cardiomyocytes. This process reduces their transcriptional levels and transitions the cardiomyocytes to a less mature state. Ultimately, this promotes cardiomyocyte proliferation and ischemia-reperfusion regeneration of the heart after injury (51). Furthermore, the role of sphingolipid metabolism in cardiac regeneration was revealed, in which sphingosine kinase (SphK), a key enzyme of sphingolipid metabolism, plays a critical regulatory role (50). SphK1 inhibits cardiac regeneration by regulating the proliferation of cardiac fibroblasts; on the contrary, SphK2 promotes the proliferation of cardiomyocytes by reversing the process of the mature cell cycle (50). It is noteworthy why SphK1 and SphK2 play diametrically opposed roles as isoenzymes. Possibly due to their different distribution locations, the specific mechanisms involved remain to be further explored.

In summary, inhibiting lipid metabolism and thus stimulating cardiomyocytes to re-enter the cell cycle may present a potential target for regenerative therapy after cardiac injury.

### 4.3 Amino acid metabolism in cardiac regeneration

Protein synthesis is necessary for cardiomyocyte growth and maturation, and increased protein synthesis inevitably leads to increased amino acid metabolism. Amino acid oxidation is also a source of cardiac ATP, of which oxidation of branched-chain amino acids (BCAAs) produces only 2% of total cardiac ATP (57), but BCAAs play an important role in regulating cardiac regeneration.

BCAAs comprise three amino acids, including leucine, isoleucine, and valine, which play an integral role in the mechanistic target of rapamycin (mTOR) signaling pathway that regulates cardiac regeneration (53). Elevated expression of glutamine as a transporter of amino acids in zebrafish and neonatal mouse cardiomyocytes activates the amino acid-driven mTOR signaling pathway, which in turn promotes mitochondrial maturation and regulates cardiomyocyte proliferation (70). In addition, high concentrations of leucine and glutamate also activate mTOR signaling and promote myocardial regeneration (70).

The Wnt signaling pathway is an important signaling pathway that regulates cardiomyocyte differentiation and cardiac regeneration (71). A study showed that neonatal mouse hearts were prepared for initiation of mTOR signaling through amino acid and Wnt/β-catenin signaling. RNA sequencing showed that Wnt/β-catenin signaling was higher in neonatal mouse hearts than in adult mouse hearts, whereas downregulation of Wnt ligand and Wnt/β-catenin signaling was found in the apical portion of P7 mice. This trend persisted into the adult stage, which is consistent with the cell cycle in cardiomyocytes exiting pattern (70). Furthermore, in the TOR-initiated state, metabolism shifts to amino acid oxidation, and zebrafish and mammalian cardiomyocytes are regenerative (70).

Thus, the metabolic state of amino acids influences the progression of the cell cycle in cardiomyocytes. However, this area has not been well studied and needs to be further explored.

### 4.4 Ketone body metabolism in cardiac regeneration

In recent years, by comparing data from the proteome and gene expression profiles of the mouse heart before and after birth, researchers have found that the activity of genes involved in ketone body metabolism changes significantly in the perinatal period. Compared with embryonic day 18.5, the expression level of a ketogenic rate-limiting enzyme, 3-hydroxy-3-methylglutaryl-CoA synthase 2 (HMGCS2), increased more than tenfold in P7 hearts, inducing a loss of cardiac regenerative capacity (72). At the same time, loss of HMGCS2 impairs postnatal cardiac development in mice, which can be rescued by lactation supplementation with ketone bodies. This is because ketone body defects inhibit lipid oxidation by inhibiting β-hydroxybutyrylation, which prevents the metabolic flow from acetyl-coenzyme A delivery to the ketone bodies (72), thereby echoing the previously discussed inhibition of cardiac proliferation and regeneration by fatty acid metabolism.

Increased ketone body metabolism is a key event in HF and pathological remodeling (73). Data from animal models and patients with HF suggest that ketone body oxidation is increased in the failing heart (74). In addition, endothelial cells of the heart are capable of oxidizing ketone bodies, which enhances cell proliferation, migration, and vascular sprouting (75). It has been shown that the beneficial effects of ketone body supplementation during cardiac hypertrophy are attributed to ketone body oxidation in cardiomyocytes and that ketone body oxidation enhances the respiratory efficiency of cardiomyocytes in failing hearts (75). The above studies suggest the importance of ketone body metabolism in the heart during pathologic cardiac remodeling.

In summary, ketone bodies inhibit cardiomyocyte proliferation after birth, possibly by regulating lipid metabolism in the neonatal heart. Ketone bodies are critical for mitochondrial maturation, the metabolic shift of mitochondria from anaerobic glycolysis to aerobic fatty acid β-oxidation, and the loss of cardiac regenerative capacity. Thus inducing a metabolic shift by inhibiting ketogenesis may promote cardiac regeneration.

### 4.5 Tricarboxylic acid cycle in cardiac regeneration

The tricarboxylic acid cycle is the metabolic center of carbohydrate-lipid-amino acid binding and the final metabolic pathway for all three nutrients. The postnatal increase in mitochondrial DNA in mice leads to a significant increase in mitochondrial ROS (42), and ROS damage cellular proteins, lipids, and DNA and induce cell cycle arrest (41, 76–78). Therefore, it is crucial to elucidate the role of the tricarboxylic acid cycle in cardiac regeneration.

Studies have shown that the hearts of neonatal mice and adult zebrafish have low mitochondrial content and complexity and lack markers for DDR; however, these markers are significantly increased in mouse hearts in the first few days of life (42). Therefore, is it possible to promote cardiomyocyte proliferation by inhibiting mitochondrial oxidation to reduce ROS production and, consequently, energy metabolism? Overexpression of the ROS scavengers N-acetylcysteine (NAC) and mitochondrial peroxiredoxin (mCAT) in cardiomyocytes was able to reduce DDR and reverse cell cycle in cardiomyocytes progression (42). In cardiomyocytes, the transcription factor (paired-like homeodomain transcription factor 2) Pitx2 promotes cardiac repair by recruiting yes-associated protein (YAP) to activate the antioxidant response to reduce ROS after cardiac injury (42). In addition, hydrogen sulfide (H_2_S) scavenges ROS and promotes the reentry of cardiomyocytes into the cell cycle, and Pei et al. inhibited myocardial proliferation and regeneration by using the inhibitor of H_2_S, DL-Propargylglycine (PAG), which promotes ROS deposition in cardiomyocytes (79). In contrast, inhibition of ROS accumulation by using sodium hydrosulfide hydrate (NaHS), a donor of H_2_S, promoted cardiomyocyte proliferation (79). The above results suggest that activating antioxidant responses after cardiac injury reduces ROS and thus promotes cardiac repair.

In cardiomyocytes, inhibition of key metabolites in the tricarboxylic acid cycle (TCA) cycle is also another strategy to inhibit energy metabolism. Bae et al. showed that malonate prolonged the proliferation window of cardiomyocytes in neonatal mice and reintroduced cardiomyocytes into the cell cycle in adult injured hearts by inhibiting succinate dehydrogenase (SDH) in the tricarboxylic acid cycle (9). By analyzing metabolites from postnatal day 0.5 to day 7 in mice, α-KG was found to rank the highest among the reduced metabolites. Subsequent injection of α-KG prolonged the window of proliferation of cardiomyocytes during cardiac development and facilitated cardiac regeneration after MI by inducing cardiomyocyte proliferation (80).

Thus, the above study reveals a strategy to inhibit energy metabolism-mediated cardiac regeneration and points to a new direction for the treatment of cardiomyopathy.

## 5 Molecular mechanisms in cardiac regeneration

**Figure 4:**
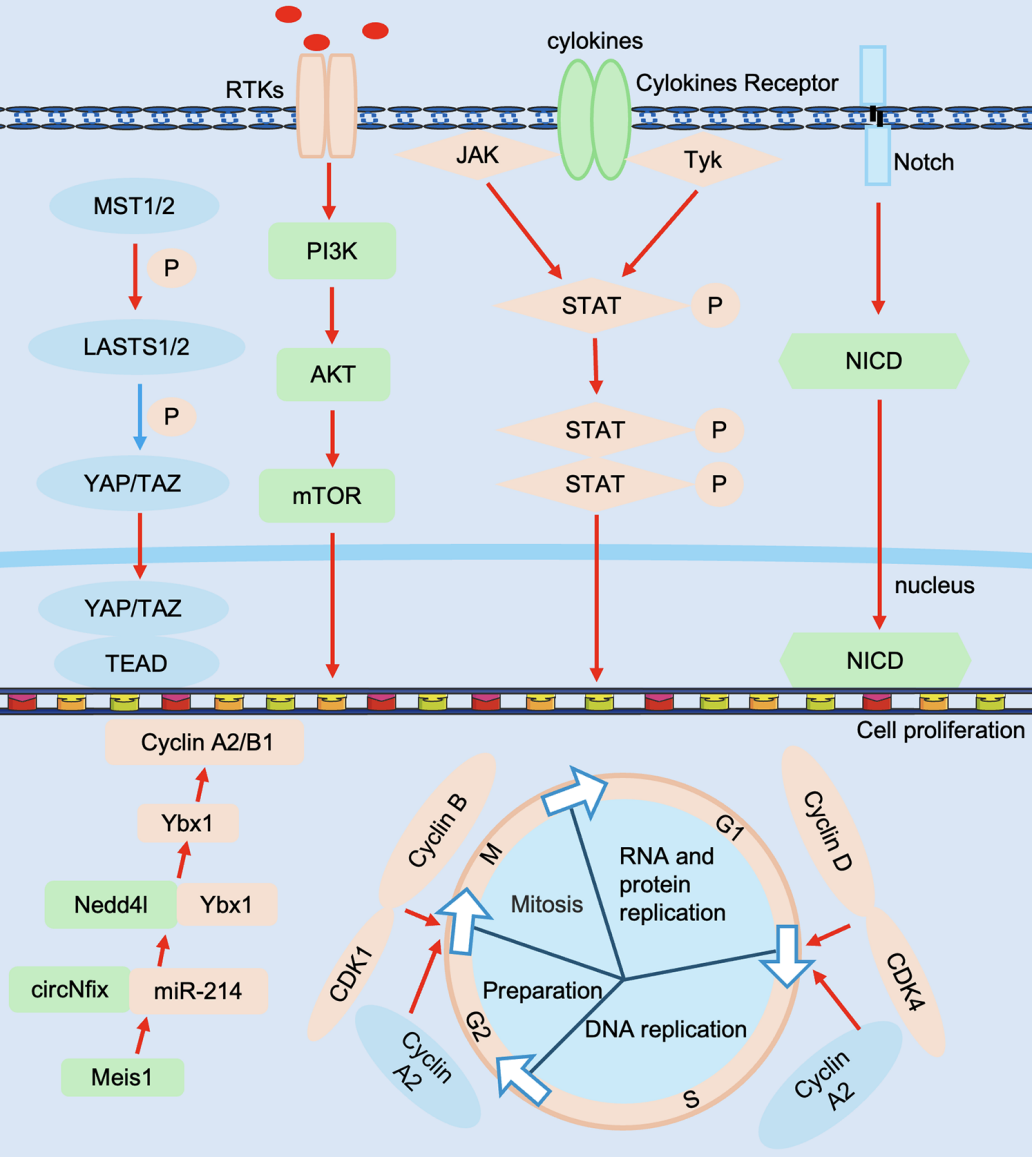
Molecular mechanisms in cardiac regeneration. Regulators include Cyclin D, CDK4, Cyclin B, CDK1, Cyclin A2, etc., non-coding RNAs include circNfix, miR-214, etc., signaling pathways include Hippo-YAP, Notch, PI3K-AkT and JAK/STAT, etc., and effectors include TEAD, NICD, etc. JAK/STAT, Janus kinase signal transducer and activator of transcription; NICD, Notch intracellular domain; PI3K-AkT, phosphatidylinositol 3-kinase-Akt; TEAD, transcriptional enhanced associate domains. The red line represents activation and the blue line represents inhibition.

Adult mammals have limited ability to regenerate their hearts after cardiac injury. However, adult zebrafish can regenerate effectively after heart injury, so it is crucial to reveal the molecular mechanisms that regulate cardiac proliferation and regeneration. Currently, more and more factors regulating cardiomyocyte proliferation have been reported, including cell cycle regulation, non-coding genes, and signaling pathways (Figure 4).

### 5.1 Cell cycle regulatory factors

Positive cell cycle regulators such as cyclin, cyclin-dependent kinase (CDK), and proto-oncogenes are highly expressed in mouse embryonic and neonatal hearts, whereas their expression tends to decrease in adult mouse hearts. In contrast, negative cell cycle regulators such as cell cycle protein-dependent kinase inhibitor (CKI) are highly expressed in adult mouse hearts and downregulated in embryonic and neonatal mouse hearts (81). Currently, promoting the reentry of endogenous cardiomyocytes into the cell cycle by modulating cell cycle factors may provide new targets or ideas for cardiac regenerative medicine.

Mammalian cell cycle activity is an important factor influencing the proliferative capacity of postnatal cardiomyocytes. Recently, researchers have focused their attention on two major protein families that regulate the cell cycle. For cell cycle proteins to play a role in regulating the cell cycle, they must bind to CDK in order to have kinase activity that phosphorylates cell cycle-associated proteins, thus playing a key role in cell cycle regulation. Thus, Cyclin D, a cell cycle protein in the G1 phase of the cell cycle, drives the G1/S phase transition by binding to CDK4. It has been shown that sustained expression of cyclin D2 drives cardiomyocyte proliferation, induces DNA synthesis, and reduces infarct size (82). Cyclin B, a cell cycle protein in the G2 phase of the cell cycle, regulates the entry and exit of cells into and out of the M phase of the cell cycle by binding to CDK1. Overexpression of the cyclin B1-CDC2 complex stimulates cell division in adult mouse cardiomyocytes, and deficiency of the cyclin B1-CDC2 complex results in adult mouse cardiomyocytes remaining in the G2/M phase and inhibits cardiomyocyte proliferation (83). Cyclin A2 promotes the transition of the cell cycle from the G1 phase to the S phase as well as from the G2 phase to the M phase. Overexpression of cyclin A2 in adult porcine hearts improved cardiac function and induced DNA synthesis and cardiomyocyte mitosis in ischemically injured mice (84). The above studies suggest that positive cell cycle proteins such as Cyclin D, Cyclin B, and Cyclin A may be potential targets for stimulating cardiomyocyte proliferation after injury.

In addition, CKI binding to CDK can inactivate the cyclin-CDK complex, thereby negatively regulating cell cycle activity and inhibiting cardiomyocyte proliferation. Recently, it has been shown that adult mouse cardiomyocytes contain a large number of CDK inhibitors, which negatively regulate the cell cycle activity of adult mouse cardiomyocytes and, therefore, inhibit the proliferation and division of cardiomyocytes (85, 86). Multiple studies have shown that CIP/KIP family members (p21, p27, and p57) are present in mature cardiomyocytes and regulate CDKs to shut down the cell cycle (87). p21 and p27 knockout mice fail to exit the cell cycle in cardiomyocytes in the G1 phase and undergo DNA replication after birth (88, 89). Mechanistically, p21/p27 binds to cyclin A/cyclin E-CDK after birth in mice and promotes cell exit from the cell cycle (88). In addition, the CDK inhibitor p27 interacts with p57 to allow cell cycle exit and differentiation to occur (90, 91). These results suggest that members of the CIP/KIP family play an important role in cell cycle exit in postnatal cardiomyocytes.

Recently, investigators found that *in vivo* overexpression of a combination of viral-mediated cell cycle regulators (CDK1/CCNB/CDK4/CCND) stimulated the proliferation of adult mouse, rat, or human cardiomyocytes. Moreover, replacing CDK1/CCNB with Wee1 inhibitors and transforming growth factor-β (TGFβ) inhibitors further facilitated the entry of cardiomyocytes into the cell cycle reduced scarring after cardiac injury area, and improved cardiac function (92). Our group's study revealed that ABRO1A (93) and TMEM11 (94) negatively regulate the cardiomyocyte cell cycle through post-translational modifications and inhibit cardiomyocyte cell cycle proliferation and cardiac regeneration. Therefore, influencing cardiomyocyte proliferation and thus promoting cardiac regeneration by modulating cell cycle regulators during cardiac growth and development is expected to be one of the future therapeutic strategies for cardiac repair.

### 5.2 Non-coding RNA

In recent years, more and more researchers have found that non-coding RNAs regulate the cell cycle in cardiomyocytes, which in turn regulate cardiomyocyte proliferation and cardiac regeneration. The following summarizes non-coding RNAs in cardiac regeneration, including microRNAs, circRNAs and lncRNAs.

microRNAs (miRs) are small non-coding RNAs that are important for suppressing gene expression and maintaining homeostasis of cardiomyocyte proliferation. A high-throughput functional screen revealed that miR-590 and miR-199a promoted the re-entry of cardiomyocytes into the cell cycle in neonatal and adult mice and facilitated cardiomyocyte proliferation and recovery of cardiac function after MI (95). It has been shown that the expression level of miR-34a is progressively upregulated after birth. Overexpression of miR-34a in the hearts of neonatal mice with MI inhibits cardiomyocyte proliferation and subsequent recovery of cardiac function. In contrast, remodeling after MI was significantly ameliorated by nucleic acid-based antimiR-34a treatment in the hearts of adult mice after MI (96). Furthermore, miR-17-92, miR302-367, and miR-294 positively regulate cardiomyocyte proliferation (97, 98), whereas miR-15 and miR-128 negatively regulate cardiomyocyte cell cycle progression (46, 99). Taken together, these studies suggest that targeting miRNAs is a promising therapeutic strategy for inducing cardiac repair processes.

circRNAs are a class of noncoding RNAs with covalent closed-loop structures. circNfix, which is predominantly expressed in cardiomyocytes, is driven by the transcription factor Meis1. It interacts with miR-214 to prompt the interaction of the E3 ubiquitin ligase Nedd4L with Y-box binding protein 1 (Ybx1), thereby inducing the ubiquitination and degradation of Ybx1. This degradation down-regulates the downstream target genes of Ybx1, Cyclin A2, and Cyclin B1, which in turn regulates cell cycle progression and thus inhibits cardiomyocyte proliferation (100). circSamd4 and circMdc1 play a role in positively and negatively regulating cardiomyocyte proliferation by localizing in the mitochondria and regulating ROS and DDR, respectively (101, 102).

After birth, lncRNA CRRL (103), lncRNA CAREL (104), lncRNA NPPA-AS1 (105), and lncRNA AZIN2-sv (106), whose expression was elevated in the heart, inhibited cardiomyocyte proliferation, whereas lncRNA ECRAR (107), lncRNA Sirt1 antisense (108), and lncRNA Snhg1 (109), whose expression is reduced in the heart, promote cardiomyocyte proliferation and regeneration. lncRNAs regulate cardiomyocyte proliferation mainly in two ways. On the one hand, it plays a role in regulating the cell cycle in cardiomyocytes through the lncRNA/miRNA/mRNA axis (103, 104, 106, 110). On the other hand, lncRNAs regulate cardiomyocyte proliferation by interacting with mRNAs and proteins to mediate chromatin remodeling and DNA repair (105, 107–109, 111).

Our research team demonstrated that lncRNA CPR recruits DNA methyltransferase 3α (Dnmt3a) to the promoter region of the Mcm3 gene, promoting methylation modification of DNA, inhibits Mcm3 expression and DNA replication induced by it, and thus inhibits cardiomyocyte proliferation (112). In summary, targeting non-coding RNA is a promising therapeutic strategy to induce cardiac regeneration.

### 5.3 Signaling pathway

The Hippo-YAP signaling pathway is a widely studied pathway for cardiac regeneration. When the Hippo pathway is activated, its kinases MST1/2 and LATS1/2 phosphorylate the nuclear transcription factor YAP. This phosphorylation prevents YAP from entering the nucleus to activate the transcription of target genes, thereby inhibiting cell proliferation. On the contrary, when the Hippo pathway is inhibited or YAP is activated, YAP enters the nucleus and binds to other transcription factors, promoting the expression of genes related to the cell cycle and proliferation (113). The downstream effector of Hippo, YAP/Transcriptional co-activator with PDZ-binding motif (TAZ), is required for postnatal cardiomyocyte proliferation, and deletion of YAP/TAZ in the mouse heart results in myocardial hypoplasia and lethality due to reduced cardiomyocyte proliferation (113). In contrast, postnatal overexpression of YAP increases cardiomyocyte proliferation (113, 114).

Wnt/β-Catenin signaling is one of the major signaling pathways regulating cardiac development and regenerative processes. The role of the Wnt pathway on myocardial proliferation is more complex, and interventions at different stages of development while targeting different parts of the pathway may yield different results. At mouse embryonic day 13.5, proliferatively active cardiomyocytes highly expressed β-catenin, and myocardial-specific knockdown of β-catenin reduced the number of proliferating cardiomyocytes. This effect was associated with changes in the level of Cyclin D2, a target gene of β-catenin (115). In addition, YAP, another key molecule in the regulation of myocardial proliferation, forms a complex with β-catenin that synergistically enhances β-catenin activity and promotes myocardial proliferation during development (116). In adult mouse cardiomyocytes, activation/elevation of β-catenin promotes cardiomyocyte proliferation and post-MI myocardial repair (63, 117).

Over the past two decades, there have also been many reports on the regulatory role of a Notch signaling pathway (Notch), phosphatidylinositol 3-kinase-Akt (PI3K-AkT), and Janus kinase signal transducer and activator of transcription (JAK/STAT) signaling pathways in cardiomyocyte proliferation. Activation of the Notch signaling pathway, a highly conserved signaling pathway in the heart, is critical for cardiomyocyte proliferation at birth and for cardiac development (118). The Notch signaling pathway promotes myocardial proliferation in neonatal individuals, and this effect is markedly attenuated in an adult mouse MI model. This phenomenon is associated with the modification of histone methylation in Notch target genes in adult cardiomyocytes, which are unable to respond to Notch signaling (119). PI3K-AKT is an intracellular signaling pathway and one of the drivers of the cell cycle. Studies on the heart have shown that activation of PI3K-AKT signaling leads to increased mitosis and higher cardiomyocyte numbers in both suckling mice and adult cardiomyocytes (120). The JAK/STAT signaling pathway is one of the signaling pathways that play a crucial role in cardiac regeneration. In zebrafish, inhibition of the JAK/STAT signaling pathway resulted in decreased cardiomyocyte proliferation and increased scar formation after myocardial injury (121). In mice, recovery from myocarditis injury is achieved by the re-entry of pre-existing cardiomyocytes into the cell cycle to proliferate through STAT3 (122).

## 6 Energy metabolism and related therapies in cardiovascular disease

Energy metabolism plays a crucial role in maintaining cardiac function, and its disorders are often closely associated with the onset and progression of various cardiac diseases. An in-depth study of changes in energy metabolism and related therapies in cardiovascular diseases is important for understanding disease mechanisms and finding effective treatments (Table 1).

**Table 1:** Energy metabolism and related therapies in cardiovascular disease.

Designation	Influence metabolic pathway	Mechanism of action	Cardiovascular disease	Type of study	Reference
TMZ	Promote glycolysis	Regulates AMPK to promote glycolysis and increase energy supply to cardiomyocytes.	Angina pectoris	*in vivo* and *in vitro*	(123, 124)
Pgrmc1	Increase in fatty acid oxidation	Reduce lipid accumulation in the heart and target the treatment of cardiomyopathy.	Cardiomyopathy	*in vivo* and *in vitro*	(125)
L-carnitine	Promotes β-oxidation of fatty acids	Regulates coenzyme A levels and reduces oxidative stress and inflammatory response.	HF, myocardial ischemia-reperfusion injury and arrhythmia.	clinical research	(126, 127)
Metformin	Promotes β-oxidation of fatty acids	Enhancement of CPT1 expression, reduction of apoptosis in cardiomyocytes.	HF	*in vivo* and *in vitro*	(128)
Rapamycin	Regulates amino acid metabolism	Inhibition of mTOR signaling pathway and inhibition of cardiomyocyte apoptosis.	MI	*in vivo* and *in vitro*	(129)
SGLT2i	Promotes amino acid metabolism	Degradation of abnormal branched-chain amino acids.	HF	clinical research	(130)
Beta-hydroxybutyric acid	Increase ketone body metabolism	Anti-inflammatory and thus heart protection.	HF	*in vivo* and *in vitro*	(131)
SGLT2i	Promotes oxidation of ketone bodies	Anti-inflammatory and anti-remodeling.	HF	clinical research	(132)
Itaconic acid	Inhibition of TCA circulation	Inhibits SDH and reduces abnormal accumulation of succinic acid.	Myocardial ischemia-reperfusion injury	*in vivo* and *in vitro*	(133, 134)

AMPK, adenylate-activated protein kinase; CPT1, carnitine palmitoyltransferase 1; HF, heart failure; MI, myocardial infarction; mTOR, mechanistic target of rapamycin; SDH, succinate dehydrogenase; SGLT2i, sodium-glucose cotransporter protein 2 inhibitors; TMZ, trimetazidine; TCA, tricarboxylic acid cycle.

### 6.1 Glycolysis and cardiovascular disease

Glycolysis is closely related to cardiac function, especially in pathological states such as MI and HF. Studies have shown that the heart increases glycolysis in ischemic states to compensate for energy deficits, although this compensatory mechanism may not be sufficient to maintain normal cardiac function. In patients with HF, increased glycolysis is often accompanied by decreased mitochondrial function, leading to reduced cardiac efficiency (135). Numerous studies have found that trimetazidine (TMZ) has significant anti-ischemic efficacy in chronic stable angina pectoris (136, 137). Its mechanism of action is mainly through the regulation of glycolysis-related enzymes - adenylate-activated protein kinase (AMPK), which in turn promotes the process of glycolysis, enabling the energy supply of cardiomyocytes to increase, thereby exerting the anti-ischemic protective effect on the heart (123).

Although TMZ has some efficacy in the treatment of heart disease, long-term use of trimetazidine may lead to drug resistance, affecting the effectiveness of treatment (138). In the future, the specific mechanism of action of trimetazidine in myocardial energy metabolism can be investigated by optimizing the dosage and regimen, further research, and exploring its interaction with other metabolic pathways. It is expected to further improve cardiac function and effectively prevent the progression of cardiomyopathy.

### 6.2 Fatty acid metabolism and cardiovascular disease

Inhibition of fatty acid oxidation in early HF is part of cardiac metabolic remodeling, but this inhibition progressively leads to mitochondrial dysfunction, which ultimately manifests itself in severe energy metabolism disturbances in advanced HF (53, 139). In addition, Pgrmc1 functions as a healthy metabolic regulator in the heart. Individuals with low cardiac Pgrmc1 expression may be more susceptible to mitochondrial damage and the progression to HF. Induction of Pgrmc1 expression increases the basal cardiac energy capacity and reduces cardiac lipid accumulation. This is achieved by enhancing fatty acid oxidation and mitochondrial respiration, which can significantly increase ATP production capacity. This enhancement is crucial for targeted therapies aimed at treating cardiomyopathies (125).

L-carnitine plays a crucial role in cardiac energy metabolism by promoting beta-oxidation of fatty acids, balanced cardiac energy metabolism, regulating coenzyme A levels, reducing oxidative stress and inflammatory responses, and protecting cardiomyocytes from damage. L-carnitine has a wide range of applications in heart diseases such as HF, myocardial ischemia-reperfusion injury and arrhythmia (126). Future studies will further reveal the mechanism of action of leucovorin and energy metabolism, optimize its clinical application, and provide more opportunities for the treatment of cardiac diseases. Metformin, a widely used insulin sensitizer in the treatment of type 2 diabetes mellitus (T2DM), not only excels in glycemic control but also has a significant positive impact on cardiovascular health. Its use in patients with HF has been shown to be safe and effective in reducing HF morbidity and mortality. The mechanism of action of this drug is mainly through the activation of AMPK, which in turn regulates lipid and glucose metabolism processes, thus optimizing the energy metabolism of the myocardium (140). Specifically, activation of AMPK promotes translocation of GLUT4 to cardiomyocyte membranes and accelerates insulin-dependent glucose uptake (141). In addition, metformin promotes mitochondrial β-oxidation of fatty acids by enhancing the expression of carnitine palmitoyltransferase 1, reducing apoptosis in cardiomyocytes, and decreasing the formation of myocardial mid- and late-phase glycosylation end products (AGEs) (128).

In summary, although Pgrmc1, L-carnitine, and metformin have potential applications in the treatment of heart disease, there are individual differences in the response of different patients to these treatments, resulting in the possibility that some patients may not derive the expected effectiveness from them (142–144). Future improvements can be made through the implementation of personalized treatment protocols, the conduct of multi-center clinical trials, and the application of cutting-edge technologies, among other multi-dimensional improvement measures. These are expected to significantly enhance the effectiveness and safety of the application of these drugs in clinical practice, thus bringing more treatment options and hope to patients with cardiomyopathy.

### 6.3 Amino acid metabolism and cardiovascular disease

Abnormalities in amino acid metabolism are closely associated with the development of several cardiac diseases. Studies have shown that patients with HF have significant changes in amino acid levels, especially in the oxidative capacity of branched-chain amino acids, leading to impaired cardiac energy metabolism (145). In addition, interactions between the gut microbiota and amino acid metabolism have been suggested to play an important role in the progression of heart disease. Amino acids metabolized by gut microbes may affect heart health by modulating cardiac function and inflammatory responses (146). Rapamycin and its analogs exert inhibitory effects on cardiomyocyte apoptosis and promote cardiomyocyte autophagy by inhibiting the mTOR signaling pathway (147). This mechanism is closely related to amino acid metabolism. In pathological conditions such as MI, rapamycin is effective in limiting the death of cardiomyocytes and thus reducing the extent of infarction. At the same time, they can also attenuate the hypertrophy of cardiomyocytes and provide relief to the process of cardiac remodeling (129). Sodium-glucose cotransporter protein 2 inhibitors (SGLT2i) have emerged as important agents in the treatment of HF and have been shown to be effective in reducing the rate of HF hospitalization and the risk of cardiovascular death (148). SGLT2i induces degradation of abnormal branched-chain amino acids in failing myocardium as an alternative source of myocardial fuel to optimize cardiac energy supply (130).

In conclusion, both rapamycin and SGLT2i have limitations in the treatment of heart disease, and rapamycin is immunosuppressive and may increase the risk of infection with long-term use (149). SGLT2i may increase the risk of urinary tract infections (150). In the future, it can be used in combination with other drugs, such as anti-inflammatory drugs. This is not only expected to reduce the risk of infection but also to further enhance the therapeutic effect on cardiomyopathy. Thus, it would provide a safer and more effective treatment option for cardiomyopathy patients.

### 6.4 Ketone body metabolism and cardiovascular disease

Ketone bodies, consisting of β-hydroxybutyrate, acetoacetate, and acetone, are metabolic byproducts known as energy substrates during fasting. In recent years, studies have shown the potential of promoting the utilization of ketone bodies as a means of preventing the progression of HF (151). In patients with HF, blood concentrations of ketone bodies increase, as does the utilization of ketone bodies within the myocardium (57, 152–154). The addition of ketone bodies has been reported to inhibit cardiac utilization of glucose in HF and increase the efficiency of energy production (155). In addition, β-hydroxybutyrate in ketone bodies inhibits the activation of NOD-like receptor protein 3 (NLRP3) inflammatory vesicles and exerts cardioprotective effects through its anti-inflammatory properties (131). SGLT2i induces a shift in the heart's primary energy source from fat to ketone bodies and promotes ketone body oxidation, a shift that not only has intrinsic anti-inflammatory and anti-remodeling effects but also provides a more efficient source of energy (132).

Beta-hydroxybutyric acid is a ketone body, and when it accumulates in the body in excess, disorders of ketone body metabolism can lead to fatal consequences such as ketoacidosis (156). Therefore, treating the disease while maintaining ketone homeostasis is also a direction that needs to be explored in future research.

### 6.5 Tricarboxylic acid cycle and cardiovascular disease

In cardiac metabolism, the TCA cycle is of particular importance. The heart is a high-energy-consuming organ that relies on efficient energy-generating pathways to maintain its contractile function. Studies have shown that the heart adjusts the activity of the TCA cycle to meet its energy requirements in different physiological and pathological states. In the case of HF, the heart enhances the utilization of ketone bodies and may compensate for the energy deficit by increasing the activity of the TCA cycle (57). In addition, intermediates of the TCA cycle, such as α-KG and succinate, are involved in regulating cardiac cell growth and survival and influencing cardiac remodeling processes (80). Itaconic acid plays an important role during ischemia as an inhibitor of SDH. It effectively reduces the abnormal accumulation of succinate during ischemia by inhibiting SDH activity (133). This reduction in ischemic succinate accumulation has a significant positive effect on ameliorating cardiac ischemia-reperfusion injury (134).

Itaconic acid is a bypass metabolite of the tricarboxylic acid cycle, and its accumulation in cells can regulate the body's immune activity through various pathways (157). However, these complex regulatory mechanisms may exhibit different effects in different cardiac pathological states, and the current understanding of their specific mechanisms of action is not sufficiently deep, which limits the wide application of itaconic acid in cardiac therapy. The specific mechanism of action of itaconic acid in different cardiac pathological states can be further investigated in the future. Through high-throughput screening and gene editing technologies, the signaling pathways and targets of itaconic acid in cells can be revealed, providing a theoretical basis for the development of more effective cardiac therapeutic strategies.

## 7 Discussion

Ischemic heart disease causes 13% of global deaths in men and up to 14% in women (158). HF occurs with an inadequate supply of cardiac energy and places a tremendous burden on the heart, which further leads to a decrease in mitochondrial oxidative capacity, an increase in glycolysis associated with glucose oxidation, and a decrease in fatty acid oxidation. The first step in cardiac regeneration is to promote the re-entry of pre-existing cardiomyocytes into the cell cycle for cell division and proliferation. In this review, we summarize the current state of research on the metabolic regulation of myocardial regeneration (Table 2), including the regulation of glucose, lipid, and amino acid metabolism for cardiac regeneration. Based on this, altering the relevant components of the metabolic pathway to reintroduce cardiomyocytes into the cell cycle and promote cardiomyocyte proliferation, which in turn enhances cardiac regeneration, is an effective therapy for the treatment of MI. The application of energy metabolism in cardiovascular disease is summarized at the end of the article (Table 1), which elucidates the relationship between energy metabolism and disease and provides a strong rationale for the transformation of energy metabolism and regenerative therapy for MI.

**Table 2:** Metabolic regulation in cardiac regeneration.

Metabolic pathway	Biomolecules	Cardiomyocyte proliferation	Mechanism of action	Reference
Glucose metabolism	GLUT1	+	Increased glucose metabolism.	(62)
	PDK	+	Encode key enzymes that regulate pyruvate metabolism and promote glycolysis.	(54)
	PKM2	+	Via anabolic pathways and β-linker proteins.	(63)
Fatty acids metabolism	CPT1	+	Inhibiting fatty acid oxidation	(51, 68, 69)
	SphK	−	SphK1 regulates cardiac fibroblast proliferation.	(50)
		+	SphK2 reverses mature cell cycle progression.	
Amino acid metabolism	BCAAs	+	Activates mTOR signaling.	(53)
	Glutamine	+	Activates mTOR signaling pathway and promotes mitochondrial maturation.	(159)
Ketone metabolism	HMGCS2	+	Inhibiting fatty acid oxidation.	(72)
TCA cycle	NAC and mCAT	+	Reduced DDR.	(42)
	Pitx2	+	Activation of Antioxidant Response to Reduce ROS after Cardiac Injury.	(42)
	H_2_S	+	Scavenging ROS.	(79)
	Malonate	+	Suppression of SDH.	(9)
	α-KG	+	H3K4me3 structural domain demethylation.	(80)

(+): upregulate; (−): downregulate.

α-KG, α-ketoglutarate; BCAAs, branched-chain amino acids; CPT1, carnitine palmitoyltransferase 1; DDR, DNA damage response pathway; GLUT1, glucose transporter proteins 1; H2S, hydrogen sulfide; HMGCS2, 3-hydroxy-3-methylglutaryl-CoA synthase 2; mTOR, mechanistic target of rapamycin; NAC, N-acetylcysteine; PDK, pyruvate dehydrogenase kinase; PKM2, pyruvate kinase muscle isoform 2; Pitx2, paired-like homeodomain transcription factor 2; ROS, reactive oxygen species; SDH, succinate dehydrogenase; SphK, sphingosine kinase; TCA, tricarboxylic acid cycle.

However, further research is needed to find solutions to questions such as, what mechanisms regulate shifts between metabolism? By what mechanisms do metabolic shifts promote cardiomyocyte regeneration after a metabolic shift? Secondly, given the potential of drugs to modulate cardiac metabolism, the development of new drugs to precisely target specific metabolic pathways will be an important research topic. Furthermore, are there other metabolic pathways involved in the regulation of cardiac regeneration in addition to the five major metabolisms discussed in this review?

Therefore, in the future, in-depth translational research can be carried out regarding the mutual transformation between myocardial energy metabolism, which can provide new targets for future clinical translation. Furthermore, it is of great significance to further explore the relationship between the myocardial regeneration mechanism and myocardial energy metabolism transformation. Myocardial regeneration is one of the key topics in regenerative medicine. The findings of the review on the inter-transformation of energy metabolism to promote cell regeneration are not only applicable to cardiac regeneration but also provide a useful reference for regeneration studies of other tissues and organs, such as nerve regeneration and liver regeneration. This has broad disciplinary implications and is of great importance for the development of the entire field of regenerative medicine. All in all, we are optimistic that human cardiac regeneration is likely to make great progress.
